# Studying neurocognitive systems for sustained attention in neurogeriatric patients: protocol of the SENSE-AGE study

**DOI:** 10.3389/fnagi.2026.1749814

**Published:** 2026-04-16

**Authors:** Lina Stagneth, Julius Welzel, Gesine Hermann, Corina Maetzler, Christian Neumann, Lea Dahl, Lily Schmeer, Henrike Knacke, Cornelia Kranczioch, Sangitam Graf, Andreas Müller, Johanna Geritz, Walter Maetzler

**Affiliations:** 1 Department of Neurology, University Hospital Schleswig-Holstein, Kiel Campus, Christian-Albrechts-University, Kiel, Germany; 2 Neurocognition and Functional Neurorehabilitation Group, Neuropsychology Lab, Carl von Ossietzky University of Oldenburg, Oldenburg, Germany; 3 Brain and Trauma Foundation Grisons, Chur, Switzerland

**Keywords:** cognition, EEG, ERP, frequency bands, neurogeriatric patients, recovery, sustained attention, vigilance

## Abstract

**Background:**

Sustained attention is a complex cognitive function required for the successful performance of tasks such as walking, cycling, driving, conversations and other prolonged tasks. Deficits of this function are associated with frailty, falls, and general cognitive decline in older adults. Sustained attention declines with age and is impaired in many neurological disorders. However, little is known about the underlying neurophysiological characteristics of sustained attention deficits in neurogeriatric patients and their interaction with other cognitive domains. Electroencephalography (EEG) provides a non-invasive and scalable method to assess neural dynamics with high temporal resolution. EEG parameters have shown promise as objective markers of cognitive dysfunction in aging and neurodegenerative conditions, but their specific relevance as surrogate markers of sustained attention in neurogeriatric patients remains unclear. Identifying reliable EEG-based surrogate markers could facilitate early detection, risk stratification, and targeted interventions. Therefore, this study aims to investigate EEG-based parameters as potential surrogate markers of sustained attention in neurogeriatric patients.

**Methods/design:**

The study “Studying Neurocognitive Systems for Sustained Attention in Neurogeriatrics Patients” (SENSE-AGE) is a prospective, explorative, observational study and will include 120 geriatric participants. At admission, participants will perform a Go-NoGo task, a Psychomotor Vigilance Task (PVT) and resting-state condition during EEG recording, using a 32-channel system. Task-based event-related potentials (ERPs) and frequency-band power will be extracted. Neuropsychological tests characterize global and domain-specific cognition and will examine associations between sustained attention, broader cognitive performance, and EEG parameters. Questionnaires will assess fatigue, sleep, health-related quality of life, and subjective cognition. A subgroup of 50 participants will be re-evaluated at the end of the inpatient stay, after 2–3 weeks of standardized geriatric complex treatment. The main hypotheses of the study are: sustained attention (i) correlates with ERP amplitude and latency; (ii) correlates with EEG power; (iii) improves after an inpatient multiprofessional complex treatment at both the task-performance and neurophysiological level.

**Discussion:**

The study protocol describes an experimental approach to investigate sustained attention in a neurogeriatric cohort combining a behavioral approach with EEG recordings. The results may help defining objective and quantitative surrogate markers of sustained attention in this vulnerable cohort.

## 1 Introduction

Attention, one of the six central cognitive domains, is crucial for daily cognitive processing, enabling a selective focus on relevant environmental and internal information while suppressing distractions (Bergius, 2009; Falkai et al., 2018). Sustained attention, or vigilance, refers to the ability to maintain focus and process relevant information over time (Klösch et al., 2022). This type of attention is essential for maintaining safety and efficiency in tasks requiring prolonged attention, such as navigating traffic, responding to environmental changes (Yanko and Spalek, 2013; Walker and Trick, 2018; Park et al., 2021), and engaging in conversations (Sturm, 2008; Häusler and Sturm, 2009). Most interestingly, its decline with age is associated with reduced mobility (O’Halloran et al., 2011; O’Halloran et al., 2014), and increased risk of falls (O’Halloran et al., 2011; Bennett Murphy et al., 2001; Ganesh and Gurumoorthy, 2021). Moreover, sustained attention is affected in various neurological conditions, including Alzheimer’s dementia (AD) (Berardi et al., 2005; Huntley et al., 2017), dementia with Lewy bodies (Collerton et al., 2003; Hamilton et al., 2024), Frontotemporal dementia (O’Keeffe et al., 2007), and Parkinson’s disease (PD), even at early stages (Pfeiffer et al., 2014; Agosta et al., 2020). For example, the prevalence of AD increases from 0.6% in individuals aged 65–69 years to more than 22% in those aged 90 years and older (Lobo et al., 2000), and mild cognitive impairment (MCI) occurs in approximately 40% of patients with PD (Dorsey et al., 2018). These disorders often manifest as reduced peripheral nerve signal quality (Anderton, 2002), slower brain processing (Salthouse, 2016), and diminished compensatory capacity (Bunzeck et al., 2024), suggesting that geriatric patients are at particularly increased risk of suffering from sustained attention deficits. The potential for treating these deficits in geriatric patients and the impact on other cognitive functions and neurophysiological patterns remain underexplored, partly reflected in limited availability of validated and clinically applicable objective markers of sustained attention (Fortenbaugh et al., 2017; Huang et al., 2023).

Although several neural networks have been identified as potentially relevant in sustained attention, including the default mode network, the frontoparietal network, the salience network, and the locus coeruleus norepinephrine system (Bonnelle et al., 2011; Smith et al., 2011; Langner and Eickhoff, 2013; Buckner and DiNicola, 2019; Slattery et al., 2022), the interaction of these systems and their contribution to age-related or clinical sustained attention deficits remain incompletely understood (Fortenbaugh et al., 2017; Huang et al., 2023). Neuropsychological and neuroimaging studies have identified specific brain regions as being associated with sustained attention, including the anterior cingulate cortex, dorsolateral prefrontal cortex, and parietal regions, predominantly in the right hemisphere (Staub et al., 2013). EEG research further indicates age-related alterations in attentional processing. Older adults may exhibit increased frontal activation during cognitive control tasks, possibly reflecting compensatory recruitment (Hong et al., 2016). In addition, reductions in P300 amplitude, prolonged latencies, and alterations in theta and alpha oscillatory dynamics have been described, suggesting changes in attentional resource allocation and processing speed (Finnigan and Robertson, 2011; Vaden et al., 2012; Staub et al., 2014). However, most evidence derives from healthy populations, and comparatively little is known about the neural dynamics of sustained attention in geriatric patients.

Several psychological theories have been proposed to explain sustained attention deficits. The resource depletion theory posits that sustained attention deficits result from a limited information processing capacity due to an individually perceived high task load (Caggiano and Parasuraman, 2004; Warm et al., 2008; Thomson et al., 2015). The mind-wandering theory postulates that a low task load results in attention drifting, thereby increasing the likelihood of mind-wandering (Randall et al., 2014). The resource control theory synthesizes the aforementioned theories, proposing that fixed attentional resources are depleted by mind-wandering and that reduced executive control over time is a consequence of this depletion (Thomson et al., 2015). All of these theories conceptualize sustained attention deficits as a form of cognitive performance loss during the sustained attention task.

The findings of Pershin et al. (2023) challenge the existing theories by suggesting that changes in sustained attention are less related to the actual performance of a sustained attention task, but are instead more closely associated with phases between subsequent trials in such task—which they describe as recovery phases. They demonstrated that electrophysiological markers such as alpha power changes within the sustained attention task, exhibited the greatest observed changes during intertrial intervals, where no response was required. This suggests that these intervals may represent phases when the brain is recovering from task-related demands (Pershin et al., 2023). We suggest that increased stress levels, which may occur when cognitive tasks require more “brain effort” in older adults, could further exacerbate sustained attention deficits by disrupting recovery phases, leading to a vicious cycle of reduced attention and increased stress (Selye, 1978; Wippert et al., 2009; McAvinue et al., 2015; Dillard et al., 2019; Luna et al., 2022; Buzsáki et al., 2003).

The combination of psychometric tests and EEG enables the concurrent evaluation of the correlation between behavioral responses and neural activity (Olbrich et al., 2015). In a general sense, EEG can be analyzed along the time domain and the frequency domain. Previous research has employed EEG parameters to investigate temporal differences in processing and responding to stimuli during sustained attention tasks. Event-related-potential (ERP) latency and amplitude parameters, as well as power changes in the alpha and theta bands, are directly related to attentional performance in older adults (Finnigan and Robertson, 2011; Vaden et al., 2012; Staub et al., 2014; Getzmann et al., 2016).

In the frequency domain, spectral analysis entails the examination of the power of predefined frequency bands, which are associated with discrete cognitive processes. For example, global alpha power and frontal-parietal theta power have been demonstrated to be associated with attention (Huizeling et al., 2021; Griggs et al., 2023). Studies on sustained attention and vigilance in younger healthy adults have indicated that there are increases in fronto-medial theta and global alpha power over time-on-task (Oken et al., 2006; Kamzanova et al., 2014; Clayton et al., 2015; Huang et al., 2023; Pershin et al., 2023), which may be indicative of increased attentional disengagement (Wascher et al., 2018) due to a reduction in information processing and cognitive resources (Pershin et al., 2023). Importantly, oscillatory dynamics have also been directly linked to behavioral performance. Persons with higher resting alpha power (∼10 Hz) showed larger late positive ERPs and better sustained attention performance (Dockree et al., 2007). However, opposite results may arise in task-related alpha modulation. Theta power showed positive correlation with task difficulty across age groups (Pergher et al., 2019).

In the time domain, ERPs reflect changes in cortical electrical activity in response to events or in relation to actions. ERPs can provide insights into the temporal course of cognitive processes. In sustained attention tasks, ERPs such as the N200 and P300 components, appear approximately 200–300 ms after a stimulus, respectively (Patel and Azzam, 2005). The N200 component is associated with stimulus detection and early attentional control (Patel and Azzam, 2005), as well as inhibitory and attentional processes (Warchoł and Zaja̧c-Lamparska, 2023). The P300 is associated to attentional processing and stimulus relevance (Polich, 2007; Staub et al., 2014), both have been associated with age-related neurocognitive functioning. Empirical evidence indicates that better sustained attention performance is associated with larger P300 amplitudes and shorter P300 and N2 latencies, whereas reduced amplitudes and prolonged latencies are linked to poorer performance (Finnigan and Robertson, 2011; Staub et al., 2014). Higher N2 amplitudes have been observed in high-performing individuals, suggesting more efficient stimulus evaluation and inhibitory control (Ramos-Loyo et al., 2004). The N200 appears to be reduced in older adults (Patel and Azzam, 2005) and in people with MCI and AD, at least in visual attention tasks (Morrison et al., 2018). The P300 also shows reduced amplitude and increased latency in older adults, indicating slower cognitive processing and reduced attentional resources (Chiang et al., 2018; Morrison et al., 2018; Chiang et al., 2024). Given the age-related changes in the N200 and P300 components (Riis et al., 2008; Van Dinteren et al., 2014; Chiang et al., 2018) and their increased prevalence in neurological disorders such as PD (Xu et al., 2022) and AD (Chiang et al., 2018), we will examine whether comparable electrophysiological patterns can be observed in a clinically heterogenous, neurogeriatric inpatient cohort, and how they relate to sustained attention performance.

Given the critical role of sustained attention in everyday life and the changes associated with aging, it is important to further investigate how sustained attention is reflected in EEG measures, including ERPs and power spectral changes in the frequency bands. Although these electrophysiological parameters have been associated with attentional processes, their clinical relevance as surrogate markers of sustained attention, particularly in neurogeriatric populations, remains insufficiently established.

Sustained attention in older populations is embedded within a broader biopsychosocial context. Sleep disturbances and poor sleep quality have been linked to reduced cognitive performance and reduced health-related quality of life (HrQoL) in aging cohorts (Costa et al., 2022; Sella et al., 2023). Affective symptoms such as depressive and anxiety states are associated with broad cognitive impairment, including reduced attentional control, executive functioning, memory and processing speed (Forbes et al., 2024). Moreover, subjective cognitive complaints (SCC) frequently diverge from objective measures but are clinically relevant for everyday functioning. Therefore, in addition to behavioral and electrophysiological measures, the present study includes assessments of fatigue, sleep quality, HrQoL and SCC to more comprehensively characterize sustained attention in aging.

The following research questions are thus defined for this study:

(i) Is there a correlation between sustained attention and ERPs, N200 and P300, of the EEG in latency and amplitude?

(ii) Is there a correlation between sustained attention and EEG power in the alpha and theta range? It will be tested if the correlations observed in the initial or final phases of the task differ from those observed over the entire task duration. Additionally, the hypothesis will be tested as to whether the correlation between SA and power varies between the performance phase of the task or rather the inter-trial (reset and relax) phases.

(iii) Does sustained attention improve after an inpatient multiprofessional complex treatment? If yes, is this reflected by changes in the ERPs/power spectrums of the EEG?

(iv) What are the associations between parameters of cognition, processing speed, mental fatigue, HrQoL and self-assessment of cognitive abilities, respectively, and ERPs/power spectrums of the EEG?

## 2 Methods/design

### 2.1 Study design

This is a prospective, explorative, observational study. Ethical approval has been obtained from the local ethics committee (University of Kiel (D 427/17; amendment of 12/2023 (Geritz et al., 2020)). The study is in accordance with the Declaration of Helsinki. All participants will receive detailed verbal and written information about the content and procedure of the study.

### 2.2 Participants

Participants will be neurogeriatric, defined as > 70 years with a primary neurological disease [participants between 50 and 69 years of age can also be included if they have at least two chronic diseases (> 6 months)] [ Sieber, 2007; Maetzler et al., 2016; Johnston et al., 2019). Other inclusion criteria are the ability to consent to the study and, if applicable, the consent of the legal representative, as well as at least 5 points on the Montreal Cognitive Assessment (MoCA, (Nasreddine et al., 2005)].

### 2.3 Procedure

**FIGURE 1:**
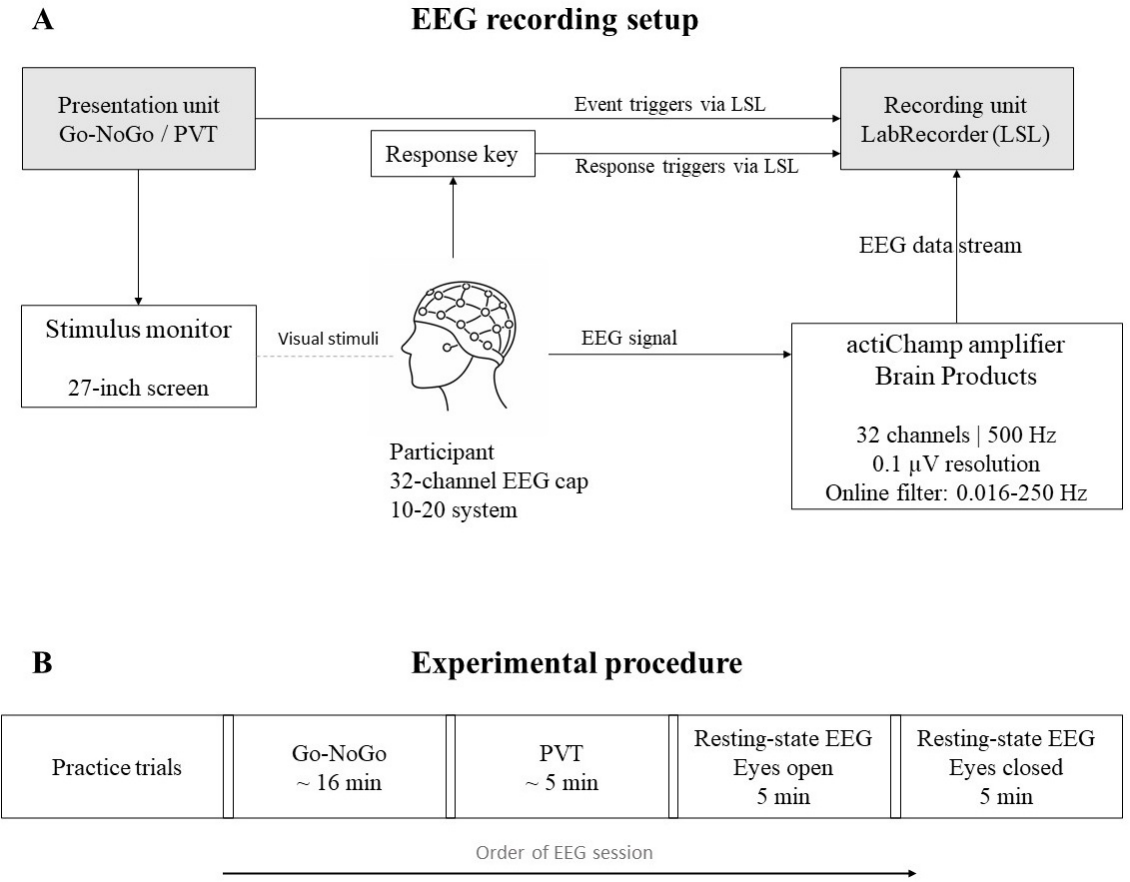
**(A)** Schematic overview of the EEG recording setup. Sustained attention paradigms (Go-NoGo task and PVT) are presented on a 27-inch monitor. Participants wear a 32-channel EEG cap arranged according to the international 10–20 system. Behavioral responses are recorded via a response key, while EEG signals are acquired using an actiChamp amplifier (Brain Products) and streamed to the recording unit via the Lab Streaming Layer (LSL). **(B)** Experimental timeline of the EEG session including practice trials, the Go-NoGo task, the PVT, and resting—state EEG recordings with eyes open and eyes closed.

**FIGURE 2:**
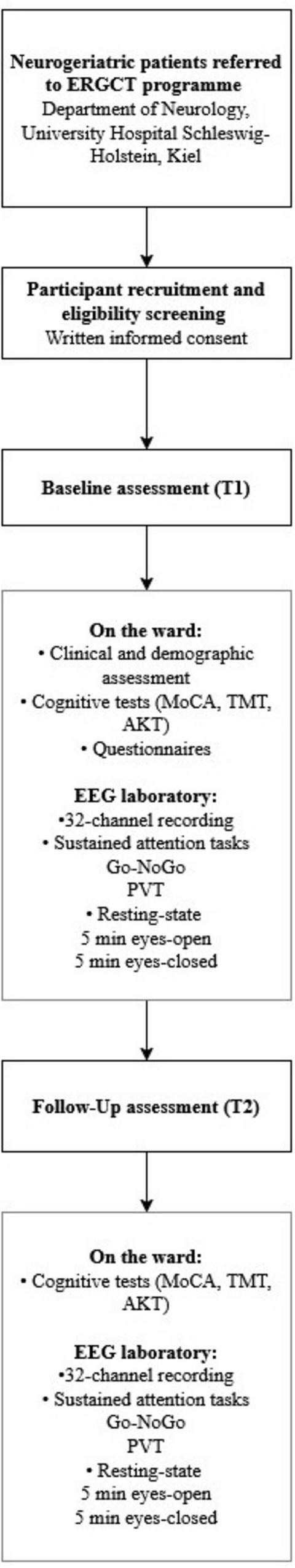
Overview of the study design and assessment procedure of the SENSE-AGE study.

Participants will be recruited at the Department of Neurology, University Hospital Schleswig-Holstein, Kiel, Germany. Participants will be enrolled within the initial 2 days of admission (T1). A subsample of 50 patients who undergo a comprehensive geriatric care (CGC) will be reassessed during the last 2 days of their inpatient stay (T2), which typically lasts 14–20 days. All participants will be provided with multidisciplinary therapy, which will be specific to their individual needs, during the course of their hospitalization. Cognitive function and attention will be evaluated using validated tests (see next section). Sustained attention and neurophysiological parameters will be evaluated using computerized tasks and EEG recordings conducted in a laboratory setting (for an overview of the EEG setup; see Figure 1). The experimental paradigms, including the Go-NoGo and Psychomotor Vigilance Task (PVT), will be presented on a 27-inch screen in a stable low-light environment. Prior to the main tasks, two practice trials of the Go-NoGo task (the first at a slower speed) and one practice trial of the PVT will be conducted. After completing both tasks, a resting-state EEG will be recorded for 5 min with eyes open and 5 min with eyes closed. An overview of the study design and assessment procedure is shown in Figure 2.

### 2.4 Clinical assessment

#### 2.4.1 Clinical and demographic data

To obtain a comprehensive description of the neurogeriatric cohort, data will be collected both in medical history and through semi-structured interviews. Functional status will be assessed using the *Activities of Daily Living* (ADL) scale (Katz, 1963), which measures basic self-care abilities. The total score range from 0 to 6, with higher scores indicating greater functional independence. The instrument has shown good reliability and validity in clinical settings, including studies reporting consistent performance characteristics in older adults (Brorsson and Asberg, 1984; Arik et al., 2015). Instrumental activities of daily living will be assessed using the IADL scale from Lawton and Brody (1969). This questionnaire evaluates more complex daily functions and total scores range from 0 to 8, with higher scores reflecting greater autonomy. The scale has shown good construct validity and sensitivity in detecting functional decline in older populations. Geriatric status will be assessed using the *Geriatrie Check* (Hobert et al., 2019), a screening tool designed to identify geriatric patients. The tool covers domains such as cognitive impairments, care dependency, frailty, and premorbid functional status. It has been validated in a cohort of hospitalized neurological patients and demonstrated good discriminative validity (Hobert et al., 2019). Diagnoses, medications, and duration and number of therapy units will be collected as covariates.

#### 2.4.2 Cognition

Global cognitive functioning will be assessed using the MoCA, a screening instrument covering multiple cognitive domains. The test yields a total score ranging from 0 to 30, with higher scores indicating better cognitive performance; a score below 26 is commonly used as a cut-off for cognitive impairment. It has good internal consistency (Cronbach’s alpha = 0.83) and a high sensitivity in the detection of MCI (90%) and AD (100%) (Nasreddine et al., 2005).

Processing speed and executive functioning will be evaluated using the Trail Making Test (TMT). TMT Part A primarily assesses visual scanning and psychomotor processing speed, whereas Part B additionally requires task switching and cognitive flexibility, reflecting divided attention and executive control. Performance is measured as completion time in seconds, with longer times indicating poorer performance. The TMT shows good construct validity and sensitivity to executive dysfunction in clinical populations (Sánchez-Cubillo et al., 2009). These instruments are included to describe global cognitive status and executive functioning and will be treated as covariates in regression models. They are not considered direct measures of sustained attention.

**FIGURE 3:**
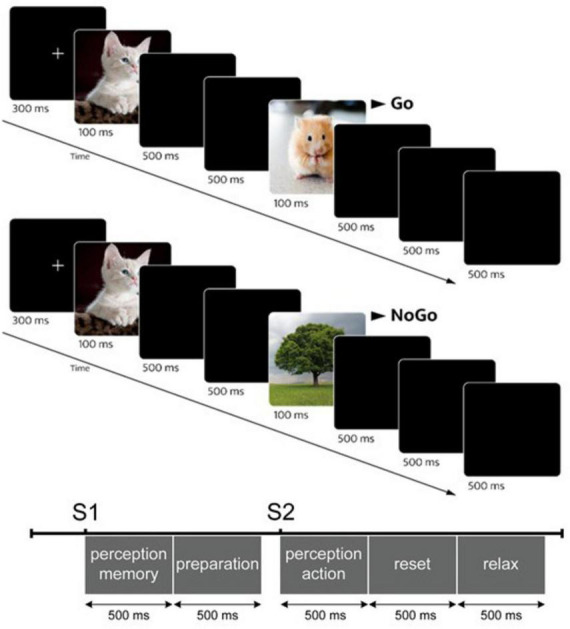
Example trials of the cued Go-NoGo task, adapted from Pershin et al. (2023). The division of the trials into distinct time windows (500 ms) allows a later analysis of the individual time segments.

Sustained attention will be measured with a 16-min cued Go-NoGo task on a computer [adapted from Pershin et al. (2023)]. Participants will see different pairs of pictures from the categories “animals,” “trees” and “fruits” (see Table 1) and have to react as fast as possible by pressing a button when two pictures with animals are shown (Go condition). In the NoGo condition, participants will see an animal as the first stimulus and a tree as the second stimulus, and have to inhibit their response (see Figure 3). The Ignore and Ignore & Novelty conditions will show two trees in a row or a tree and a fruit. No response will be required in these conditions. The presentation duration (100 ms), the interstimulus interval (1,000 ms) and the inter-trial interval will be controlled and the stimulus presentation will be randomized. Sustained attention performance in the Go-NoGo task will be quantified using several behavioral parameters. Median reaction time (RT) for correct responses in Go trials will serve as an index of sustained attentional performance. In addition, omission errors (failure to respond to Go stimuli, calculated as the proportion of missed Go trials) will be analyzed as indicators of attentional lapses, and commission errors (responses during NoGo trials, calculated as the proportion of false responses) as indicator of inhibitory control failures. Furthermore, discriminability (*d*’) will be calculated based on signal detection theory [*d*’ = Z(hit rate) – Z(false alarm rate)] to provide a bias-independent measure of sustained attention performance (Fortenbaugh et al., 2015).

**TABLE 1:** The four different conditions of the Go-NoGo task.

Condition	Stimulus 1	Stimulus 2
Go	Animal	Animal
NoGo	Animal	Tree
Ignore	Tree	Tree
Ignore and novelty	Tree	Fruit

In addition to the cued Go-NoGo task, sustained attention will be assessed using the PVT. In the PVT (computerized version), participants will press a button as quickly as possible when a red number appears on the screen. The PVT is a simple reaction time task that isolates vigilance and is particularly sensitive to attentional lapses and fluctuations in alertness, as reflected by reaction time variability and lapses.

To capture attentional stability over time during the Go-NoGo and PVT, intra-individual variability of reaction times (e.g., standard deviation of RTs across Go trials) will additionally be examined, as increased variability has been associated with reduced sustained attention (Hultsch et al., 2002; West et al., 2002; Macdonald et al., 2006).

The *Alters-Konzentrations-*Test (AKT) was developed as a paper-pencil test for older adults and measures the ability to stay vigilant and concentrated. Including the AKT allows comparison of the experimental data with conventional neuropsychological metrics commonly used in geriatric settings. Retest reliability is good (*r* = 0.75–0.89).

Although the Go-NoGo, PVT, and AKT differ in temporal structure and cognitive demands, all three paradigms require sustained attention over time and are therefore conceptualized as complementary operationalizations. Their joint inclusion allows examination of convergent validity across computerized and conventional assessment formats.

Participants’ perception of their ability to pay attention is measured using the FEDA (*Fragebogen erlebter Defizite der Aufmerksamkeit*) questionnaire (Theml and Romero, 2001), a 27-item self-report questionnaire assessing perceived attentional difficulties in everyday situations. The instrument comprises three subscales reflecting cognitive distractibility and mental slowing, fatigue and slowing in practical activities, and reduced attentional activation. Items are rated on a five-point Likert scale (1: “very often”, 2: “often”, 3: “sometimes”, 4: “rarely”, 5: “never”), with lower sum scores indicating more pronounced perceived deficits. The FEDA discriminated between patients with very mild Alzheimer’s dementia and matched healthy controls with an accuracy of 82% (Theml and Romero, 2001).

#### 2.4.3 HrQoL, behavior, sleep and subjective assessment of cognition

In addition to objective behavioral and electrophysiological measures of sustained attention, the study assesses health-related and psychological factors that are known to influence cognitive functioning in older adults. Sustained attention in neurogeriatric populations is embedded within a broader biopsychosocial context, in which affective symptoms, sleep disturbances, fatigue, and perceived cognitive decline may modulate both behavioral performance and neural dynamics. These variables will therefore be included (i) as potential confounders in regression models and (ii) as exploratory correlates of sustained attention performance and EEG parameters (Research Question iv). Participants will be asked to complete the following digitized questionnaires on a tablet.

##### 2.4.3.1 Health-related quality of life

HrQoL will be assessed using the *European Quality of Life 5 Dimensions 5 Level Version* (EQ 5D-5L) (Herdman et al., 2011). It records HrQoL on the current day in the domains of mobility, self-care, usual activities, pain/discomfort and anxiety/depression. In addition, participants can rate their general health on a visual analog scale (“0” = worst health, “100” = best health they can imagine).

##### 2.4.3.2 Affective symptoms and fatigue

Symptoms of depression will be assessed using the *Depression im Alter* scale (DIA-S) (Heidenblut and Zank, 2010), which consists of 10 items related to the past 14 days. Additionally, anxiety and depressive symptoms will be measured using the *Hospital Anxiety and Depression Scale* (HADS) (Petermann, 2011). The HADS consists of 14 items (7 anxiety, 7 depression), each rated on a 4-point scale (0–3), resulting in subscale scores ranging from 0 to 21. Internal consistencies for both subscales are typically around α = 0.80, and the two-factor structure (anxiety/depression) has been well established. Cut-offs allow classification into normal, borderline and clinically relevant symptom levels.

Fatigue will be assessed using the *Functional Assessment of* Chronic Illness Therapy—Fatigue (FACIT-F) (Montan et al., 2018). The FACIT-F is a validated multidimensional instrument assessing fatigue severity over the past seven days on a 5-point Likert scale. A score ≤ 30 has been widely accepted as a clinical threshold for fatigue (Piper and Cella, 2010). Mental fatigue has been shown to impair sustained attention, increase reaction time variability, and alter frontal EEG activity (Wascher et al., 2014; Möckel et al., 2015; Kunasegaran et al., 2023).

##### 2.4.3.3 Sleep quality

Sleep quality over the past 4 weeks will be assessed using the *Pittsburgh Sleep Quality Index* (PSQI) questionnaire (Hinz et al., 2017). The PSQI captures seven components, including subjective sleep quality, sleep latency, sleep duration, sleep efficiency, sleep disturbances, use of sleep medication, and daytime dysfunction, which are combined into a global score. Higher scores indicate poorer sleep quality. State sleepiness at the time of testing will be assessed using the *Karolinska Sleepiness Scale* (KSS), a single-item 9-point scale measuring momentary subjective sleepiness (Geiger Brown et al., 2014).

##### 2.4.3.4 Subjective cognitive complaints

Subjective cognitive complaints will be assessed using the *Complainer Profile Identification questionnaire* (CPI) (Lubitz et al., 2018). The CPI comprises 17 items rated on a 5-point frequency scale (“never” to “very often”) and differentiates complaints across the three domains memory, executive function and attention, which can also be presented separately. Previous work shows that SCCs are strongly influenced by affective symptoms and psychosocial factors (Reid and MacLullich, 2006; Smit et al., 2024), and do not necessarily align with objective cognitive performance (Slavin et al., 2010). Including the CPI and the FEDA therefore enables investigation of whether EEG-based markers of sustained attention are more closely related to objective performance or to perceived cognitive difficulties.

#### 2.4.4 Electroencephalography

EEG will be recorded in a laboratory environment to reduce and standardize external influences, such as light and noise. We will record EEG using a 32-channel layout connected to an actiChamp amplifier (BrainVision Brain Products GmbH). Electrodes will be placed according to the 10–20 system and online data collection will be done with an amplitude resolution of 0.1 μV and a sampling rate of 500 Hz, with online filters of 0.016–250 Hz. We aim for electrode impedances below 15 kΩ. EOG activity will be recorded using a single dedicated EOG electrode, placed below the left eye. Data will be recorded via the LSL protocol using the Lab Recorder (Kothe et al., 2024). Frequency bands will be analyzed during a subsequent 10-min resting period, divided into 5 min with eyes closed and 5 min with eyes open.

### 2.5 Data analysis and statistical approaches

Non-time series data, such as questionnaires, will be partially digitally collected and managed using the Research Electronic Data Capture (REDCap) tool hosted at the University of Kiel (Harris et al., 2009; Harris et al., 2019). REDCap is a secure, web-based software platform designed to support data capture for research studies, providing (1) an intuitive interface for validated data capture; (2) audit trails for tracking data manipulation and export procedures; (3) automated export procedures for seamless data downloads to common statistical packages; and (4) procedures for data integration and interoperability with external sources. Statistical analyses will be performed using JASP (currently Version 0.18.3) (JASP Team, 2024). We will use common descriptive and inferential statistics, as well as appropriate non-parametric statistics, to summarize the baseline data (Fortenbaugh et al., 2015). We will test for normality with Shapiro-Wilk test and Q–Q plots. To assess time-on-task effects, paired *t*-tests or Wilcoxon signed-rank tests will be used to compare reaction times and errors between the first and last quarters of the tasks (Pershin et al., 2023). In addition, correlation analyses (Pearson or Spearman) will be performed to assess the relationship between the number of trials and reaction time. Multiple linear regression will be used to analyze the influence of confounding factors (e.g., age, gender, depression, and cognitive function) on task scores.

Paired *t*-tests or Wilcoxon signed-rank tests will be used for pre-post comparisons to detect changes in clinical variables, as well as behavioral and EEG measures of sustained attention.

#### 2.5.1 Controlling for confounding variables

To ensure the validity of our findings, we will control for potential confounding variables such as age (Shearer et al., 1989; Mizukami and Katada, 2018; Tröndle et al., 2023), cognitive status (Soikkeli et al., 1991; Baker et al., 2008), medication use (Centorrino et al., 2002; Blume, 2006; Geraedts et al., 2018), and mental disorders, such as depression, anxiety, and mental fatigue (Greco et al., 2021; Lin et al., 2021; Brush et al., 2022). We will perform an analysis of covariance (ANCOVA) to evaluate the effect of cognitive task performance on EEG components, specifically the N200 and P300. The peak amplitudes and latencies of these components will serve as dependent variables, while performance metrics from the PVT and Go-NoGo tasks will serve as independent variables. The model will identify the impact of task performance on EEG outcomes while controlling for the influence of potential confounding variables.

#### 2.5.2 EEG preprocessing

EEG data processing will be conducted using MNE (v1.3.1) (Gramfort, 2013) in Python (3.10). To ensure optimal ICA decomposition quality, a copy of the raw data will be high-pass filtered at 1 Hz (FIR, zero-phase), re-referenced to the common average, and used exclusively for ICA fitting. Noisy channels will be identified and interpolated using the PyPREP package (v0.4.2; (Bigdely-Shamlo et al., 2015)), followed by ICA decomposition. Artifactual independent components (e.g., ocular, cardiac, and muscle artifacts) will be identified using the ICLabel classifier (Pion-Tonachini et al., 2019) and confirmed by visual inspection. The resulting ICA weights and the list of excluded components will subsequently be applied to a separate copy of the raw data bandpass filtered at 0.1–30 Hz. This approach preserves slow neural activity below 1 Hz that is relevant to ERP morphology, following the recommendations of Winkler and colleagues (Winkler et al., 2015). The resulting cleaned, bandpass-filtered (0.1–30 Hz) data form the basis for all subsequent ERP and frequency-band analyses.

#### 2.5.3 Event-related potentials

This study will examine the relationship between various ERP components and sustained attention, with emphasis on amplitude and latency. Peak measures are defined as the maximum amplitude of a signal within a specified time window, along with the corresponding time point (latency) at which this peak amplitude occurs. Specifically, we will extract peak measures for the N200 and P300 components. The N200 and P300 will be analyzed in the context of the PVT and the Go-NoGo task, focusing on target responses as well as the occurrence of omission and commission errors.

#### 2.5.4 Frequency-band analysis

For the frequency band analysis, the focus will be on analyzing the alpha and theta frequency bands during attentional tasks such as the Go-NoGo and PVT. This analysis aims to determine if there are correlations between task performance and changes in frequency bands, particularly if increased alpha band activity is associated with decreased task performance. In addition, we will examine whether the most significant changes in these frequency bands occur during the performance phases of the tasks or during inter-trial periods. To this end, we utilize a classification of trials into the following segments, as proposed by Pershin and colleagues (Pershin et al., 2023). They divided the trial phases *post hoc* into the five segments “perception/memory,” “preparation,” “perception/action,” “reset” and “relax.” Figure 3 illustrates an example sequence of a single trial. For the frequency band analysis, power spectral density (PSD) will be calculated using Welch’s method (Welch, 1967) with further processing by the FOOOF (Fitting Oscillations and One Over F) package (Donoghue et al., 2020) to extract power values in predefined frequency bands (delta 1–4 Hz, theta 4–8 Hz, alpha 8–13 Hz, and beta 13–30 Hz).

## 3 Discussion

Although attention is one of the six central cognitive domains and a fundamental function required for many other cognitive processes (Falkai et al., 2018), and studies on sustained attention across different age groups show significant differences in performance and neural dynamics in older adults (McAvinue et al., 2012; Langner and Eickhoff, 2013; Fortenbaugh et al., 2015), research on this topic in the neurogeriatric field is rare. The study presented here will investigate whether EEG has the potential to provide objective and quantitative surrogate markers of sustained attention in neurogeriatric patients. In particular, we aim to determine possible EEG changes during active task performance and during inactive (“recovery”) phases. These findings may help to elucidate pathomechanisms underlying sustained attention deficits, with potential therapeutic implications.

The strength of our proposed study lies in investigating a typical neurogeriatric population at a university hospital, covering a wide range of neurological conditions and cognitive abilities. Furthermore, the protocol employs a comprehensive multimodal assessment strategy. Sustained attention will be examined using three complementary paradigms, the cued Go-NoGo task, the PVT, and the AKT, thereby capturing sustained attention across computerized reaction-time paradigms as well as a conventional paper–pencil format commonly used in geriatric settings. Within these tasks, sustained attention will be quantified not only by mean reaction times but also through omission and commission errors, and intra-individual reaction time variability, reflecting complementary aspects of vigilance performance.

On the electrophysiological level, the combination of ERP measures (N200, P300) and spectral power analyses allows for a differentiated characterization of neural dynamics associated with sustained attention (Polich, 2007; Clayton et al., 2015; Liu et al., 2020). Given that EEG recordings in older and multimorbid patients are often associated with increased movement-related and physiological artifacts (Urigüen and Garcia-Zapirain, 2015; Geraedts et al., 2018), particular attention will be paid to data quality and preprocessing procedures. Artifact correction using ICA in combination with automated component classification (ICLabel) will be applied, alongside visual inspection, to ensure careful handling of artifact-prone recordings. The integration of behavioral, electrophysiological, clinical, and self-reported measures strengthens construct validity and enables the examination of sustained attention within a clinically meaningful and ecologically valid framework. Important covariates that are known to impact treatment success and quality of life will also be considered. Moreover, in a subgroup, we will examine potential changes in sustained attention performance over 2–3 weeks of complex geriatric treatment.

From an analytical perspective, the study is designed to go beyond descriptive comparisons and to examine associations between behavioral and electrophysiological markers using correlational and multivariate approaches. We expect sustained attention performance indices (e.g., reaction time variability) to correlate with ERP parameters, particularly P300 amplitude and latency, as well as with frontal theta and global alpha power. Multiple regression analyses will allow us to determine whether EEG markers explain variance in sustained attention performance beyond global cognitive status (MoCA), age, and affective symptom burden. Exploratory moderation analyses using regression models with interaction terms will examine whether fatigue, depressive symptoms, or sleep quality influence the relationship between behavioral performance and neural markers. This analytical framework allows differentiation between electrophysiological parameters that reflect sustained attention specifically and those that may represent broader cognitive or affective influences.

In conclusion, this study aims to provide a structured and multimodal characterization of sustained attention in neurogeriatric patients. More specifically, it aims to evaluate the potential of EEG-derived parameters as clinically meaningful surrogate markers for the detection and monitoring of sustained attention deficits in this vulnerable cohort.

### 3.1 Limitations due to missing control group

The absence of a control group precludes any conclusion regarding the extent of sustained attention deficits due to mere age-related changes in sustained attention. In addition, several practical challenges may arise when implementing EEG protocols in a neurogeriatric inpatient population. Increased movement, tremor, reduced tolerance for prolonged testing, and medication effects may introduce variability into electrophysiological measures. Fatigue-related fluctuations during the computerized sustained attention tasks may also differentially affect early vs. late task phases. The clinical heterogeneity of the cohort, while increasing ecological validity, may lead to increased variance and reduced statistical power for specific subgroup effects. Moreover, the observational design limits the interpretability of results concerning potential causal inference between sustained attention, EEG parameters, and psychosocial variables.

For the subsample of participants who will be measured twice, the absence of a control group precludes a definitive conclusion regarding the extent to which any observed changes over the study period can be attributed to the complex geriatric treatment, as opposed to other potential influences, such as an extended stay in hospital or care facility in general. Additionally, even considering the generally good retest reliabilities of resting-state and task-based EEG measures, it will not be possible to causally attribute the observed changes in ERPs or power to the treatment of the participants (Näpflin et al., 2008; Vázquez-Marrufo et al., 2020; Popov et al., 2023).

## 4 Conclusion and outlook

Taken together, this protocol presents a multimodal framework to investigate sustained attention in neurogeriatric patients by integrating behavioral, electrophysiological, and psychosocial measures. By examining associations between EEG-derived parameters and behavioral sustained attention performance, the study aims to clarify the neural correlates of sustained attention in a clinically relevant cohort. The findings may inform future longitudinal and interventional research targeting attention deficits in neurogeriatric care.
